# Reproducing a decision-making network in a virtual visual discrimination task

**DOI:** 10.3389/fnint.2022.930326

**Published:** 2022-08-10

**Authors:** Alessandra Trapani, Francesco Jamal Sheiban, Elisa Bertone, Serena Chiosso, Luca Colombo, Matteo D'Andrea, Francesco De Santis, Francesca Fati, Veronica Fossati, Victor Gonzalez, Alessandra Pedrocchi

**Affiliations:** NearLab, Department of Electronics, Information and Bioengineering, Politecnico di Milano, Milan, Italy

**Keywords:** NEST, decision-making, network model, reproducibility, working memory, neurorobot

## Abstract

We reproduced a decision-making network model using the neural simulator software neural simulation tool (NEST), and we embedded the spiking neural network in a virtual robotic agent performing a simulated behavioral task. The present work builds upon the concept of replicability in neuroscience, preserving most of the computational properties in the initial model although employing a different software tool. The proposed implementation successfully obtains equivalent results from the original study, reproducing the salient features of the neural processes underlying a binary decision. Furthermore, the resulting network is able to control a robot performing an *in silico* visual discrimination task, the implementation of which is openly available on the EBRAINS infrastructure through the neuro robotics platform (NRP).

## 1. Introduction

Given the complex hierarchical organization of the brain, a holistic understanding of the biological mechanisms underlying brain functions still pose a huge challenge. For instance, deciding between two alternatives is a basic but essential task the brain has to perform, and very little is known about its biological implementation. Computational neuroscience models can partially address the quest for an algorithmic comprehension of the different neural mechanisms. A simple biophysically-based spiking network model was presented in Brunel et al. (2001) and Wang (2002), reproducing salient characteristics of decision-correlated neural activity observed *in vivo*, such as the slow time integration of sensory stimuli (how evidence is accumulated for choosing between the alternatives) and the winner-take-all mechanism (underlying the formation of categorical choices). The model developed by the authors was intended to reproduce studies on visual motion discrimination tasks (Britten et al., 1996; Shadlen and Newsome, 2001; Roitman and Shadlen, 2002), in which primates are trained to fixate a screen displaying randomly-moving dots and make a binary decision based on the perceived point cloud movement direction, reporting their choice with a saccadic eye movement. Britten et al. (1996), Shadlen and Newsome (2001), and Roitman and Shadlen (2002) performed multiple experiments using different levels of coherence, intended as the percentage of coherently moving (e.g., with the same direction) dots, and observing the relative changes in the behavioral outcomes.

These physiological and micro-stimulation studies helped shed light on the anatomical and functional basis of decision-making processes in the brain, suggesting that neurons in the middle temporal (MT) and medial superior temporal (MST) areas (MT/MST) encode the visual motion stimulus, while the decision process itself occurs downstream in the posterior parietal cortex (LIP area). During visual motion discrimination tasks, the LIP area receives inputs from MT/MST and guides the subsequent saccadic eye movements (Shadlen and Newsome, 1996, 2001). In particular, the LIP area shows a slow ramping activity during stimulus presentation and persistent activity throughout a delay between the stimulus onset and the primate's saccadic response. Furthermore, activity in LIP is correlated with the decision process and neither with stimuli exhibition nor with motor responses, as it presents a faster ramping activity with higher coherence even if the motor output is the same (Shadlen and Newsome, 2001). Thus, pieces of evidence suggest that LIP represents the biological substrate in the brain in which decisions are formed.

In order to reproduce the LIP activity during visual motion discrimination tasks, Wang (2002) designed a recurrent balanced excitatory-inhibitory (E-I) network of spiking neurons dividing the excitatory neuron population into two sub-populations, with synapses within the same population stronger than those across populations. Thanks to this connection scheme, the model is able to describe the winner-take-all competition mediated by feedback inhibition and the corresponding attractor dynamics: the two excitatory sub-populations compete with each other depending on the external input they receive, and the population with stronger activity suppresses the other, aided by the inhibitory population. When this happens, the balance is broken, and the network activity shifts toward an attractor state. Moreover, by including slow synaptic reverberation mediated by NMDA receptors *via* a specific time-constant in the synaptic input current, the model is also able to capture the elevated persistent activity during the delay period after the stimulus onset (e.g., the primate's working memory). Thus, the model offers a solid computational explanation of how the brain is able to carry out decision-making tasks based on simple neuron models and their connectivity.

However, any scientific model is bound to be proved wrong or incomplete and replaced by a more accurate one as our understanding of physical phenomena increases. For such replacement to happen, models must be reproducible to be tested, evaluated, criticized, and ultimately modified, replaced, or even rejected. If a model cannot be reproduced, different research groups will have to produce similar models from scratch to get to similar (or slightly more advanced) conclusions each time, preventing incremental knowledge from being acquired and consolidated (Topalidou et al., 2015). It is therefore of paramount importance for computational neuroscientists to be able to reproduce and replicate studies published in the literature, notwithstanding the years passed since they were initially carried out. The concept of reproducibility has indeed witnessed a growing interest among the scientific community over recent years, leading to the proposal of 10 standard guidelines for reproducible computational research (Sandve et al., 2013).

Although “replicability” and “reproducibility” bear similar meanings, these terms have different definitions, according to the Association for Computing Machinery: a study is said to be “replicable” if its measurements can be obtained with stated precision by a different team using the same measurement procedure, the same measuring system, under the same operating conditions, in the same or different location on multiple trials (e.g., independent groups can obtain the same result(s) using the study's original artifacts); a study is instead “reproducible” if measurements can be obtained with stated precision by a different team, a different measuring system, in a different location on multiple trials (e.g., an independent group can obtain the same result(s) using artifacts which they develop completely independently) (Plesser, 2018).

The aim of this work is to reproduce the cortical decision-making network presented in Wang (2002) in a more efficient and systematic way using the neural simulation tool (NEST), a computer program for simulating large heterogeneous networks of point neurons (Gewaltig and Diesmann, 2007). NEST is especially suited for modeling large networks of spiking neurons to investigate the dynamics, size, and structure of neural systems; moreover, one of the key principles of NEST is replicability itself, as it is designed to generate analogous results independently of the hardware a spiking network simulation is run on Brette et al. (2007).

By reproducing the decision-making network model using NEST, this study also implements a saccadic eye-movement task *in silico* reconstruction using the NeuroRoboticsPlatform (NRP). The NRP is a software tool that allows running digital experiments embedding NEST-based spiking neural networks in virtual robots operating through a 3D physical simulator (GAZEBO) (Falotico et al., 2017). The platform lets users handle the flow of information between the two simulators (NEST and GAZEBO) by writing custom Python scripts called “transfer functions” that encode sensory stimuli as network inputs and transform the firing rates of different neural populations into motor commands. Thus, other than probing the reproducibility of the computational model proposed in Wang (2002), the present study investigates its translational value in the field of virtual embodied neuroscience (Mascaro et al., 2020).

The key contribution of this work can be summarized as follows: we provide a new software implementation for the well-established model proposed by Wang in 2002 and make the simulation code easily accessible and usable for the scientific community (on github). This new implementation takes advantage of the implementation in NEST and improve the simulation of the network by keeping the computational load low enough to embed the network in a virtual neurorobot and simulate the behavioral task used to study the decision-making process in primates. As a consequence, we have embedded the spiking network in an *in silico* experiment that closely replicates the behavioral task performed *in vivo*. To our knowledge, this is the first time a spiking neural network model for the decision-making process has been observed inside *in silico* experiments that closely replicate the behavioral task performed *in vivo*.

## 2. Materials and methods

### 2.1. Network model

**Figure 1:**
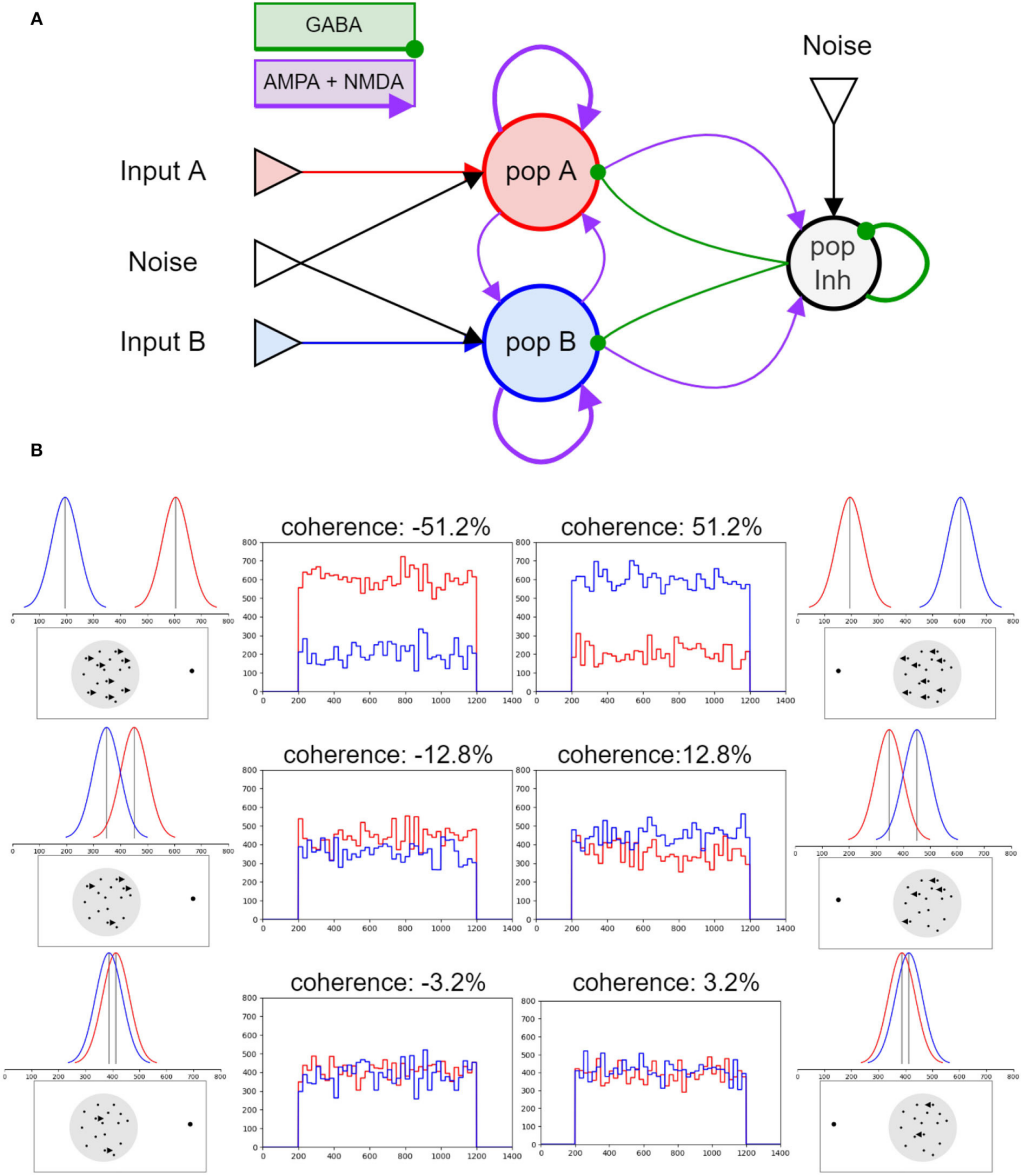
Network architecture and stimulus model: **(A)** Schematic representation of the model proposed by Brunel et al. (2001). The two neural populations that contain excitatory pyramidal cells are represented in red, pop A responsive to stimulus A, and blue, pop B responsive to stimulus B. The gray circle represents the interneurons, i.e., the inhibitory population. All inputs, stimuli and noise, are Poisson generator represented by the triangles in the picture. A bigger circle represents numerous populations, while thicker connectors represent higher connection weights. **(B)** Stimulus model: here six levels of coherence are reported. How the inputs vary in time (central plots), the Gaussian distributions from which the values are sampled every 25 ms (upper lateral plots), and the correspondent representation of the physical visual stimulus (bottom lateral images).

The cortical network modeled here is composed of two subpopulations of excitatory pyramidal neurons, each of them selective to one of the two Poisson generators that encode the directional stimulus. An inhibitory population gives shared feedback to the two excitatory populations and allows competition between them. The network architecture described in Figure 1 is taken from Brunel et al. (2001).

As in Wang (2002), the network is composed of 2,000 neurons, 80% of which belong to the excitatory populations (*A* = 800;*B* = 800 in Figure 1) and 20% to the inhibitory interneurons (*I* = 400). Different from Brunel et al. (2001) and Wang (2002), where only a small subset (*f* = 0.15) of the excitatory populations are respectively activated by the stimulus, in our network, all pyramidal (excitatory) neurons receive external inputs. Although such a difference may introduce a saturated spiking activity, this alteration is compensated by the fact that our network is not fully connected as in Wang (2002), but comprises a more physiological connection probability between the neural groups.

### 2.2. External inputs

All neurons receive a Poisson background noise whose firing rate is drawn from a Gaussian distribution of mean μ_*noise*_ and variance σ_*noise*_, during all the simulation time. The stimuli are modeled by stochastic Poisson generators, one for each motion direction. They are representative of the MT neurons response that increases with the stimulus coherence *c* in its preferred direction and decreases with *c* for stimulus in the opposite direction (Britten et al., 1996).

The firing rate of the two Poisson generators randomly changes during the stimulus presentation (updated every 25 ms) following a Gaussian distribution, with mean μ_*A*_ or μ_*B*_, respectively, and SD σ_0_. As in Wang (2002), we use a symmetrical linear relationship μ_*A*_ = μ_0_*0.5−(0.5*c) and μ_*B*_ = μ_0_*0.5+(0.5*c) (Figure 1). Therefore, if the coherence level is positive, for instance, 3.2%, the stimulus delivered on population B would be stronger than the stimulus delivered on population A, μ_*B*_ = 771*0, 516 = 397, 8*Hz* and μ_*A*_ = 771*0, 484 = 373, 2*Hz*.

### 2.3. NEST implementation

We performed the simulations on common laptop computers (8–16 GB of RAM, Intel(R) Core(TM) i7 CPU @ 1.80GHz) running Linux OS, as NEST allows computationally efficient implementation. The simulation real-time factor was on average 0.2 s, meaning that to simulate 3 s of the experimental protocol, with a simulation step of *dt* = 0.1 s, it took approximately 15 s.

#### 2.3.1. Neuron model

Both pyramidal neurons and interneurons are modeled as leaky integrate-and-fire neurons with exponential-shaped postsynaptic currents (PSCs) (Tsodyks et al., 2000). To account for the different receptor dynamics, we used “*iaf_psc_exp_multisynapse*” model (Brette and Gerstner, 2005; Schutter, 2010) that allows setting different decay time constant τ_*syn*_. The following equation describes the membrane potential:


(1) $$\begin{array}{l} C\frac{dV}{t}=-g_{L}(V-E_{L})+g_{L}\cdot \text{Δ}t\cdot exp((V_{m}-V_{th})\text{Δ}t)\\ \;+I_{syn_{tot}}(V,t)-w+I_{e} \end{array}$$

Where


(2) $$\begin{array}{r} I_{syn_{tot}}\left(V,t\right)=\underset{i}{\sum }g_{i}\left(t\right)\left(V-E_{rev,i}\right) \end{array}$$

The synapse *i* is excitatory or inhibitory depending on the value of *E*_*rev, i*_. The spike adaptation current *w* and the external current *I*_*e*_ are set to zero. All the neuron model parameters shown in Table 1, are the same reported as in Wang (2002) since they replicate the cortical neurons' biophysical properties.

**Table 1:** Network and neuron models parameters.

*V*_*m*_	–70[mV]	*J*	0.04[mV]	τ_*syn*_ noise	5[ms]
*V*_*th*_	–50[mV]	μ_0_±σ_0_	772 ± 48[Hz]	τ_*syn*_ AMPA	2[ms]
*V*_*reset*_	–55[mV]	μ_*noise*_±σ_*noise*_ to exc	7719 ± 38[Hz]	τ_*syn*_ NMDA	100[ms]
τ_*m*_ excitatory	20[ms]	μ_*noise*_±σ_*noise*_ to inh	5789 ± 28[Hz]	τ_*syn*_ GABA	5[ms]
τ_*m*_ inhibitory	10[ms]	*w*_+_	1.7	τ_*delay*_ noise	0.5[ms]
*C*_*m*_ excitatory	500[pF]	*w*_−_	0.8	τ_*delay*_ AMPA	0.5[ms]
*C*_*m*_ inhibitory	200[pF]	*w*_*NMDA*_	4.25	τ_*delay*_ NMDA	2.5[ms]
τ_*ref*_ excitatory	2[ms]			τ_*delay*_ GABA	0.5[ms]
τ_*ref*_ inhibitory	1[ms]				

#### 2.3.2. Connectivity

The synapses within the neural populations are all static synapses defined by a weight, a delay time constant, and a connection probability ϵ (“*pairwise_bernoulli*” rule in NEST, as defined in Lefort et al., 2009). As shown in Table 1, all synapses are characterized by a latency of 0.5 [*ms*] except the ones mediated by NMDA receptor that have a time delay constant of 2.5 *ms*, to account for the 2 *ms* rise time of NMDA currents, that cannot be otherwise specified as a parameter of the neuron model.

The weights of the synapses are defined by the amplitude of the postsynaptic receptor-specific currents multiplied by a dimensionless parameter *w* that represents the strength of the potentiated (*w*_+_) or depressed (*w*_−_) synapses and remain fixed throughout the simulation. The values were chosen after tuning the network to reach an adequate firing rate level and avoid saturation. As in Wang (2002), the recurrent connections mediated by the NMDA receptor (*w*_*NMDA*_ = 4.25) and connections projecting from the excitatory population to the inhibitory one and viceversa (*w*_+_ = 1.7) are strongly potentiated. Strong recurrent connections within a neural group are required in order to generate persistent self-sustained activity. On the other hand, the connection between the two excitatory populations is weakened by a factor of *w*_−_ = 0.8.

The synaptic currents are normalized by $J_{norm}^{receptor}$ such that the amplitude of the postsynaptic potential is equal to the parameter *J*. For the computation of $J_{norm}^{receptor}$, refer to Brunel (2000).

Excitatory connections between and within populations are mediated by AMPA and NMDA receptors; inhibitory synapses on pyramidal cells and recurrent inhibitory connections are mediated by GABA receptors. Connection with Poisson generators for the background noise and the stimuli are mediated by AMPA receptors only.

### 2.4. NRP experiment reconstruction

Custom experiments on the NRP require users to organize the experimental setting, choose a robotic subject among different available templates, and design the transfer functions interfacing the brain model with the environment.

**Figure 2:**
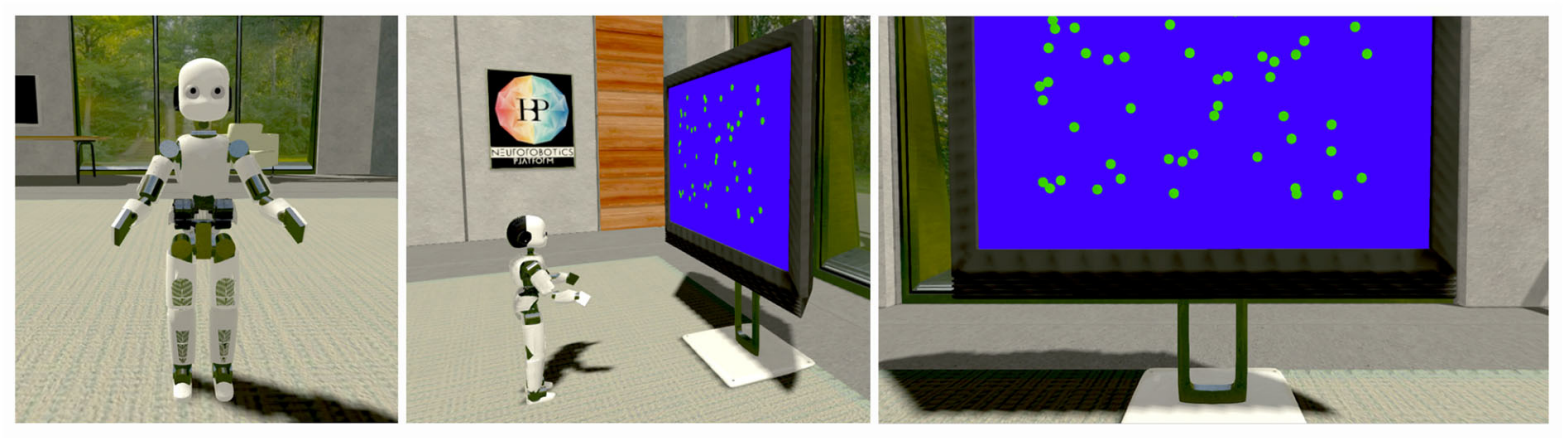
Neurorobotics platforms (NRP) experimental setting: In the proposed NRP implementation of a visual discrimination task, an iCub humanoid robot (*left*) is placed in front of a screen displaying fifty random moving green dots (*center*), occupying most of the robot's field of view (*right*). During each trial, the robotic subject is required to fixate the screen and report the perceived coherent motion of the point cloud with a saccadic eye movement, as in the corresponding primate experiments reported in the literature (Britten et al., 1996; Shadlen and Newsome, 2001; Roitman and Shadlen, 2002).

To replicate the task proposed in Britten et al. (1996), an iCub humanoid robot (Sandini et al., 2007) is placed in a virtual room in front of a screen displaying 50 random moving green dots Figure 2. The brain model embedded in the robot is the same network described in Section 2.1 downscaled to 1,000 neurons to reduce its computational load due to the NRP functional requirements (consuming more resources than NEST).

The simulated task is organized as follows: at the beginning of each trial, the green dots are distributed across a screen occupying most of the robot's field of view; then, the dots are moved around the central area of the screen (receptive field) for 5 s with an arbitrary coherence value, varying at each repetition. Randomly moving dots follow linear trajectories with different orientations and directions, while coherently moving dots all move from left to right following a straight horizontal line. This way, a transfer function encodes the intensity of the sensory stimulus computing the optical flow of the moving dots from the frames captured by the robot's cameras. The optical flow, defined as the apparent motion of individual pixels on the image plane (Turaga et al., 2010), is computed using the Lucas-Kanade registration algorithm (Lucas and Kanade, 1981), and the coherence value is encoded as the ratio between the number of horizontal flows (represented as two-dimensional vectors) and the total number of flows detected. This value is then used to set the firing rate of the network inputs as described for the Poisson generators in Section 2.2.

A second transfer function converts the firing rate of the two excitatory populations to saccadic motor commands: the robot's cameras are moved to the left or the right by a quantity proportional to population A and population B firing rates, respectively. Finally, 5 s after the stimulus onset, the moving dots are stopped and cleared from the screen before starting a new trial. A 10 s interval separates each trial; during this interval, any external input to the network ceases, and its activity return to the baseline state.

## 3. Results

To test the network's performance and compare the results obtained in Wang (2002), we ran 1,000 trials over the different coherence levels. We employed the same coherence levels as in Wang (2002): population B was stimulated using coherence values of 3.2, 6.4, 12.8, 25.6, and 51.2%, while the values for population A were –3.2, –6.4, –12.8, –25.6, and –51.2%.

### 3.1. Reproduced relevant features

**Figure 3:**
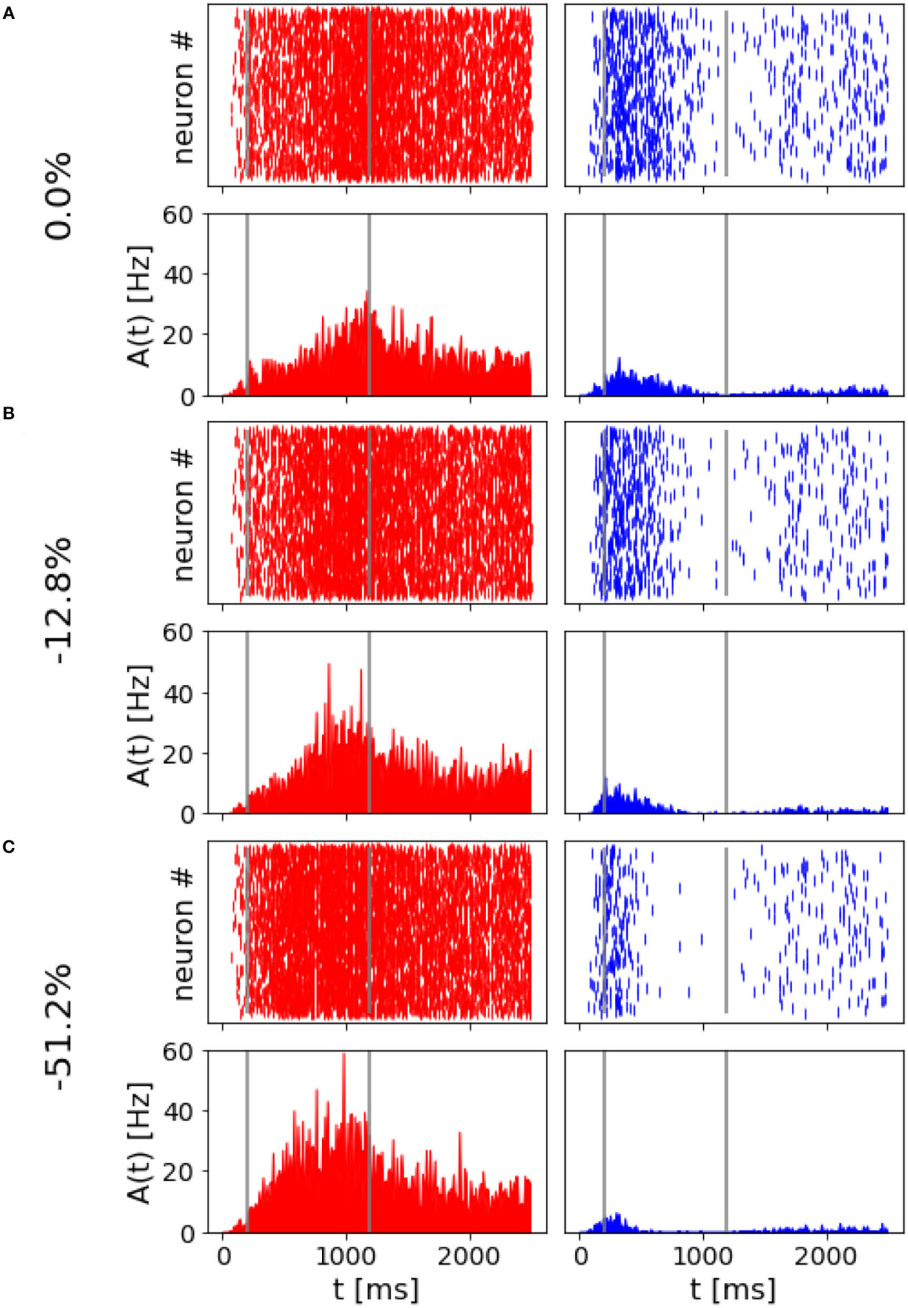
Network activity at different coherence levels: Population A response is reported on the left panels, population B response on the right. In **(A–C)**, top panels report the raster plot for the spiking activity for all neurons, while the bottom plots report frequency rates of the two populations. Gray vertical lines indicate the start and end of the stimulus delivery. **(A)** Simulation output for a trial where 0.0% coherence level was provided and population A wins. **(B)** Trial with 12.8% of stimulus coherence. **(C)** Trial with 51.2% of stimulus coherence.

**Figure 4:**
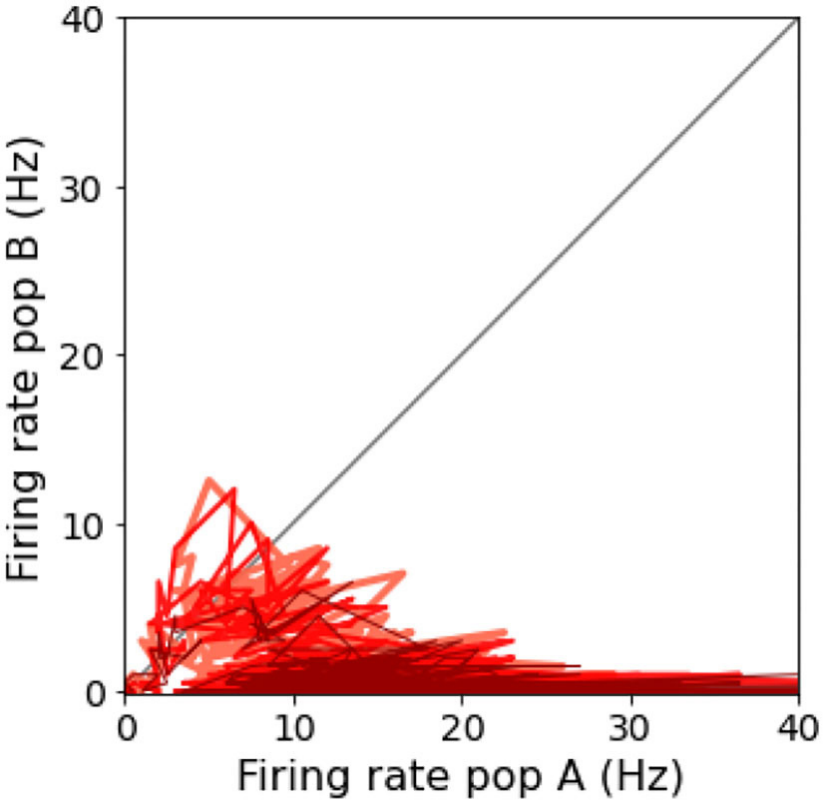
Decision space: Decision space representation of the three trials report in Figure 3. Darker lines represent a higher level of coherence: light red, 0.0%; red, 12.8%; and dark red, 51.2%.

The spiking network model presented in this study is able to mimic the salient features of the LIP neurons firing patterns during delayed visual motion discrimination task as reported in Shadlen and Newsome (2001) and modeled by Brunel et al. (2001) and Wang (2002). In Figure 3, we select three trials where population A wins over population B when stimulated at three different coherence levels. Even at low stimulus coherence, the network dynamic leads to one of the two attractor states, showing an elevated persistent activity of the winning population during a prolonged period after the stimulus ends, which suppresses the activity of the other neural group. This slow progressing winner-takes-all competition relies on the recruitment of the inhibitory neurons, which show a ramping activity similar to the one of the winning population (not shown). At an increased level of coherence in the stimulus, the winning population firing rate is slightly higher (Figure 3C). Moreover, from the decision space represented in Figure 4, we can see that the network's dynamic reaches the attractor state faster when stimulus coherence is higher. In contrast, there is a longer random walk around the decision space diagonal at low coherence, meaning that it takes more time for the two populations' activities to diverge. This reflects the physiological behavior of the cortical network, which accumulates faster evidence about the input, the stronger the input signal. Therefore, we observe a relationship between the coherence level and the steepness of the slope in the activity of the winning population that is almost identical to the one reported in Wang (2002).

#### Effect of recurrent excitation and NMDA mediation

In Wang (2002), the author found that the strong synaptic excitation on the recurrent connections is the main drive for neural integration and the slow reverberation depends mainly on the slow dynamics of the NMDA receptors. First, the network proved to decrease the recurrent excitatory weights, from *w*_+_ = 1.7 to *w*_+_ = 1.4 and observed that the ramping activity was limited in time, and there was no persistent activity after the stimulus. At low coherence, the network could not reach any attractor states, being the firing activity of the two populations at the same intensity.

**Figure 5:**
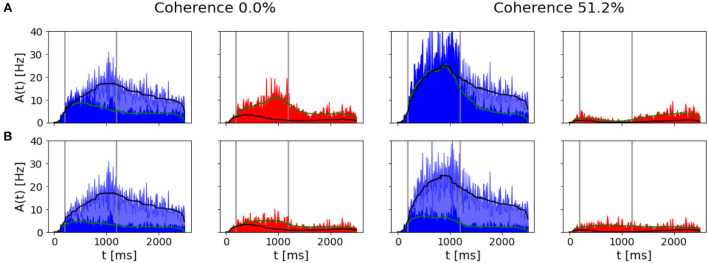
Effects of strong recurrent connection and NMDA slow reverberation: **(A)** Simulation output for decreased recurrent weights. **(B)** Simulation output without NMDA slow dynamics. We reported in blue population B activity in the altered simulations, light blue the standard simulation output. For population A we reported in red the activity in altered simulation and light red the standard response.

We performed the same testing (Figure 5A) and found that no categorical decision can be made in the case of a low coherence level since the two populations share the same activity. With a stronger stimulus being delivered, we also find that the mnemonic ability of the network is destroyed (no persistent activity).

When we simulate the network removing the slow time constant of NMDA receptor (τ_*syn*_NMDA=τ_*syn*_AMPA see Table 1), we no longer observe a silencing of the opponent population, while in Wang (2002), the network is still able to reach an attractor state even if this condition does not last after the stimulus ends (no persistent activity). As we can see from Figure 5B, when a 51.2% stimulus coherence is given as input, the network is not able to integrate the stimulus for a sufficiently long time interval to ramp up and reach a firing rate strong enough to silence the competing population. This is due to the fact that we remove the slow dynamic component of the neuronal response, given originally by the NMDA receptor, therefore only a fast response is allowed, resulting in the inability of the neuronal population to sum up different contributions in a progressive way (ramping activity).

### 3.2. “*Coin toss*” neuronal response

**Figure 6:**
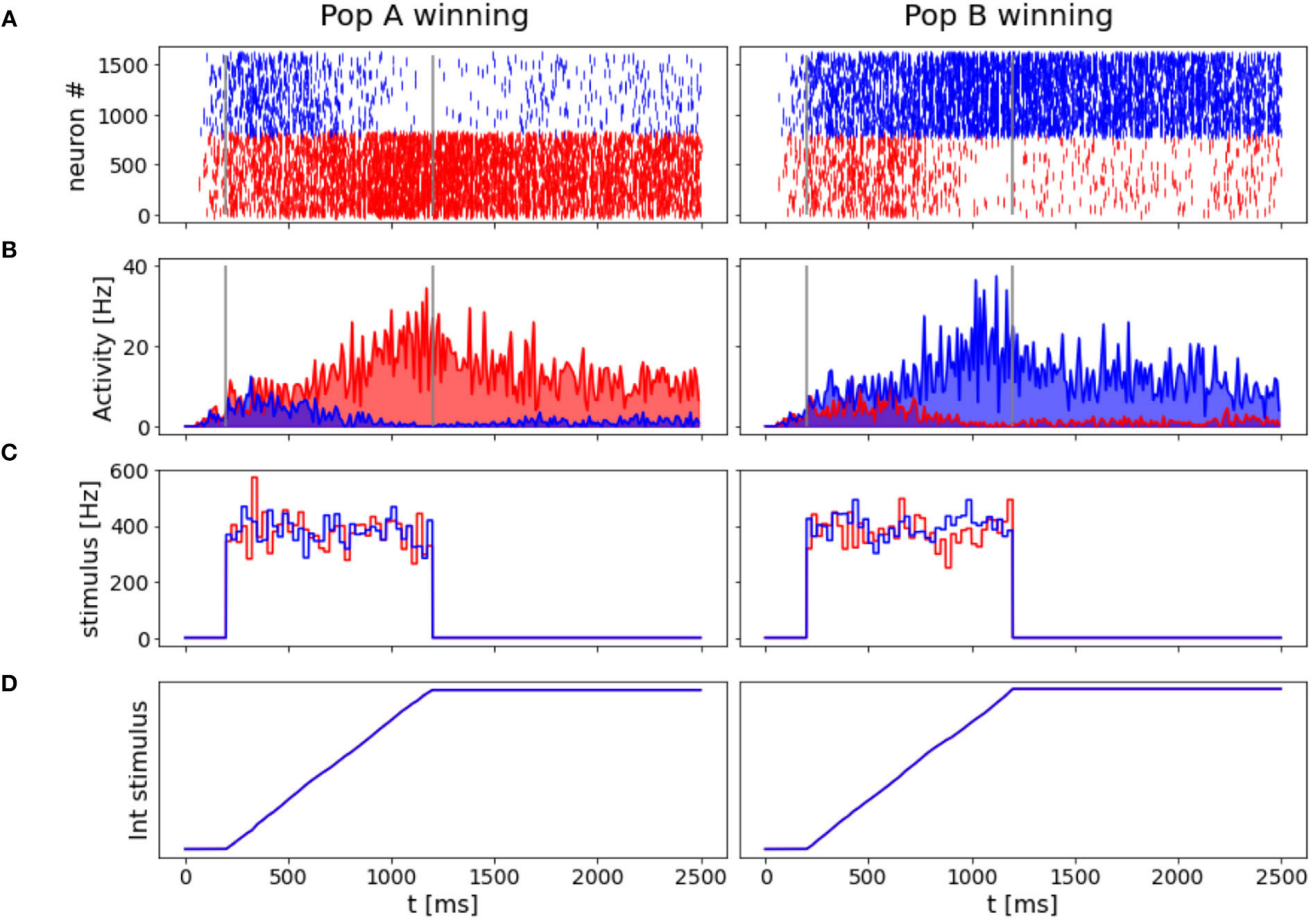
“Coin toss” decision: Here represented two trials where 0.0% coherence level is given to the network. In the left panels, population A wins over B, on the right, population B wins over A. **(A)** Raster plots for all excitatory neurons in the network color coded with respect to the population they belong to: in red Population A, in blue population B. **(B)** Firing rates across time for the two neuronal groups. **(C)** Input stimuli time course. In blue the stimulus is given to population B, in red the stimulus is given to population A. **(D)** The time integral of the two inputs.

Another interesting property of the network is its ability to take a decision even when no preferred direction for stimulus is given. In Figure 6, we reported the results for two simulation trials at zero coherence, along with the stimulus level.

In accordance with Wang (2002), when we deliver a stimulus with 0.0% coherence level, the two neural groups share the same level of activity during the initial part of the stimulus and slowly start to diverge at the end of the stimulus interval. Eventually, one of the two populations shows a prevalent self-sustained activity able to suppress the other population, i.e., taking a decision. We investigate the “nature” of the random decision, arguing whether it would be caused by the variability of the input stimulus or by the noisy nature of the background input, as done by Wang (2002). Thus, we run 1,000 additional simulations removing the stochastic fluctuations in the input (σ_0_ = 0) and compare the percentage of choices made by the network. We obtain that in 478 over 1,000 trials (47.8 vs. 42.0% in the trials where σ_0_=) the network chooses the preferred direction for A, and the remaining 522 (52.2 vs. 58.0% in the trials where σ_0_=) it chooses the preferred direction for B. Therefore, we also conclude that the main source of variability in the decision process and its outcome is due to stochastic fluctuations of a background noise (Churchland et al., 2011) here modeled as Poisson inputs to the network, that could be biologically associated with the variability of the afferent inputs of MT neurons to the LIP area (Wang, 2002).

### 3.3. Network's performance as a function of input coherence

As already mentioned, we run 1,000 trials over different levels of coherence and compare the percentage of the correct choice of each population with the Weibull function fitted over (Wang, 2002) data:


(3) $$\begin{array}{r} \%correct=1-0.5\times \exp\left(-{\left(c/\alpha \right)}^{\beta }\right), \end{array}$$

**Figure 7:**
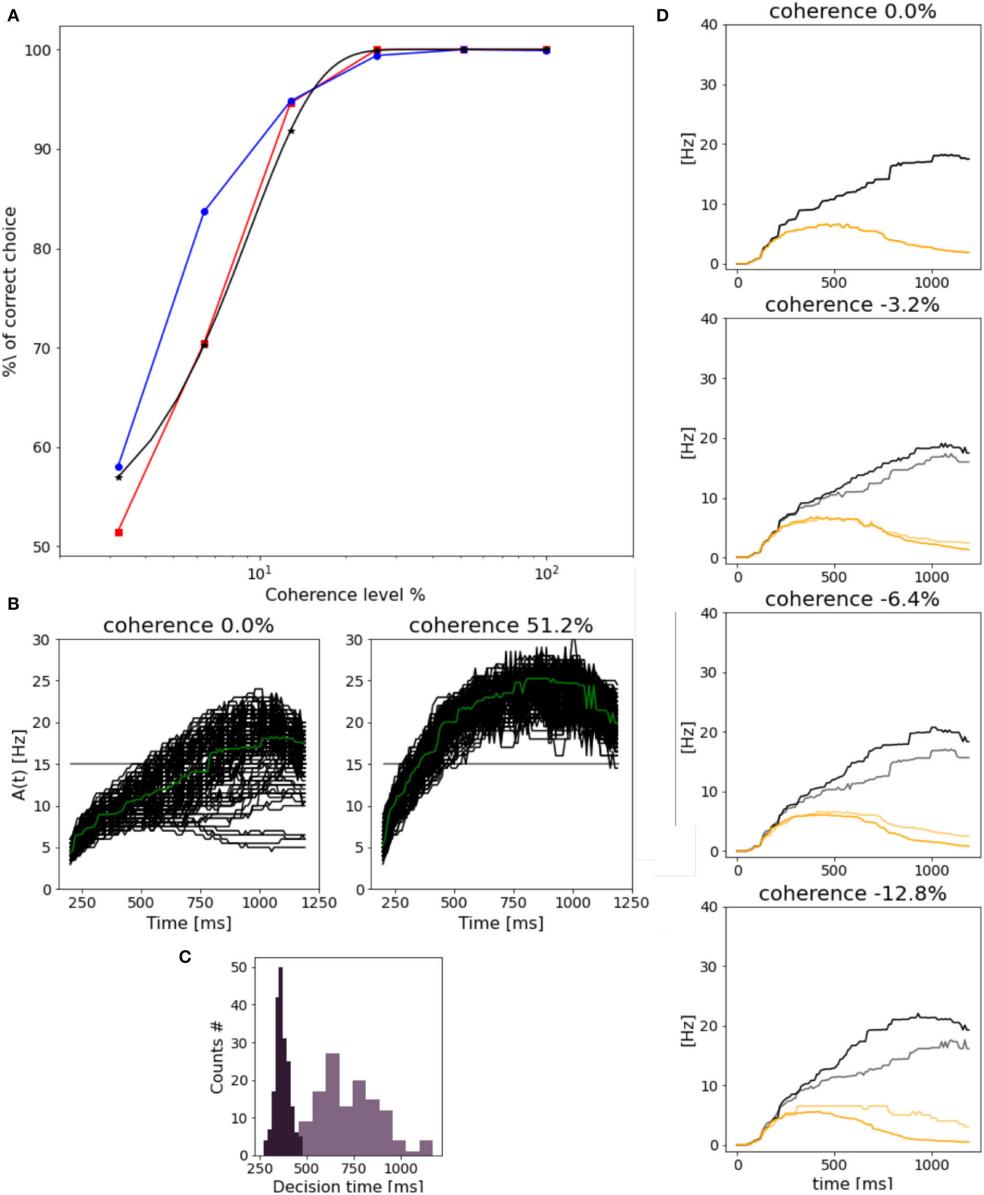
Network performance and reaction time: **(A)** Neurometric function reported as the percentage of the correct choice. In blue for population B, in red for population A and in black the weibull fit as reported by Wang (2002). **(B)** Response of population B (median filter applied to the original trace) in trials with 0.0% (left) and 51.2% (right) stimulus coherence. Green line represents the mean along trials. **(C)** Dark purple plot is the decision time histogram for trials where the network was stimulated with 51.2% coherence. Light purple histogram, for trials with 0.0% coherence level. **(D)** Evolution of population B response for four different coherence levels. Black curves (correct trials): population B wins over A and the stimulus is in the preferred direction for population B. Gray curves (error trials): population B wins but the stimulus is the non-preferred direction for population B. Orange curves (correct trials) population B loses over A, and a non-preferred stimulus for B is delivered. Light orange (error trials) population B loses over A, even if the stimulus was in the preferred direction for B.

Wang (2002) reported α = 9.2 and β = 1.5, being close to the value for psychometric functions reported in experimental studies (Shadlen and Newsome, 2001; Roitman and Shadlen, 2002). As shown in Figure 7A, we get comparable results for the response of population A, but slightly different for population B's preferred choice.

We also assess the reaction time at different coherence levels. In Figure 7B, we compare the reaction time of population B at 0.0 and 51.2% stimulus coherence. As in Wang (2002), we set a threshold of 15 Hz to measure the time at which we consider the decision to be made. In this way, we can define the reaction time as the time interval from the stimulus start until the activity of the population reaches 15 Hz. We can notice that the response to 0.0% is slower, and a higher variance with respect to the response to the stimulus at 51.2% is faster and similar in each trial. Indeed in Figure 7C, we can observe that reaction times for high stimulus coherence tend to be smaller and distributed less broad with respect to reaction times for 0.0% coherence level.

Reaction times do not depend only on stimulus coherence but also on the “correctness” of choice. In Figure 7D, we compare the time evolution of neuronal response for population B at four coherence levels, in four different cases. (1) when the correct choice has been made (black curves), e.g., population B responds to its preferred stimulus; (2) when the population B responds to a non-preferred stimulus (gray curves); (3) when population A wins over population B in response to a preferred stimulus for B (orange curves); (4) when population A wins over population B when the preferred stimulus for A is given to the network (yellow curves). In error trials, cases (2) and (3), the neuronal activity of population B increasingly differs from the one shown in correct trials (1) and (4), when stimulus coherence increases. This could be due to the fact that while in correct trials population B response changes accordingly to the levels of coherence, responses in error trials do not change, being the response time and the frequency level are almost the same for different stimuli.

### 3.4. Stimulus duration and decision reversal

**Figure 8:**
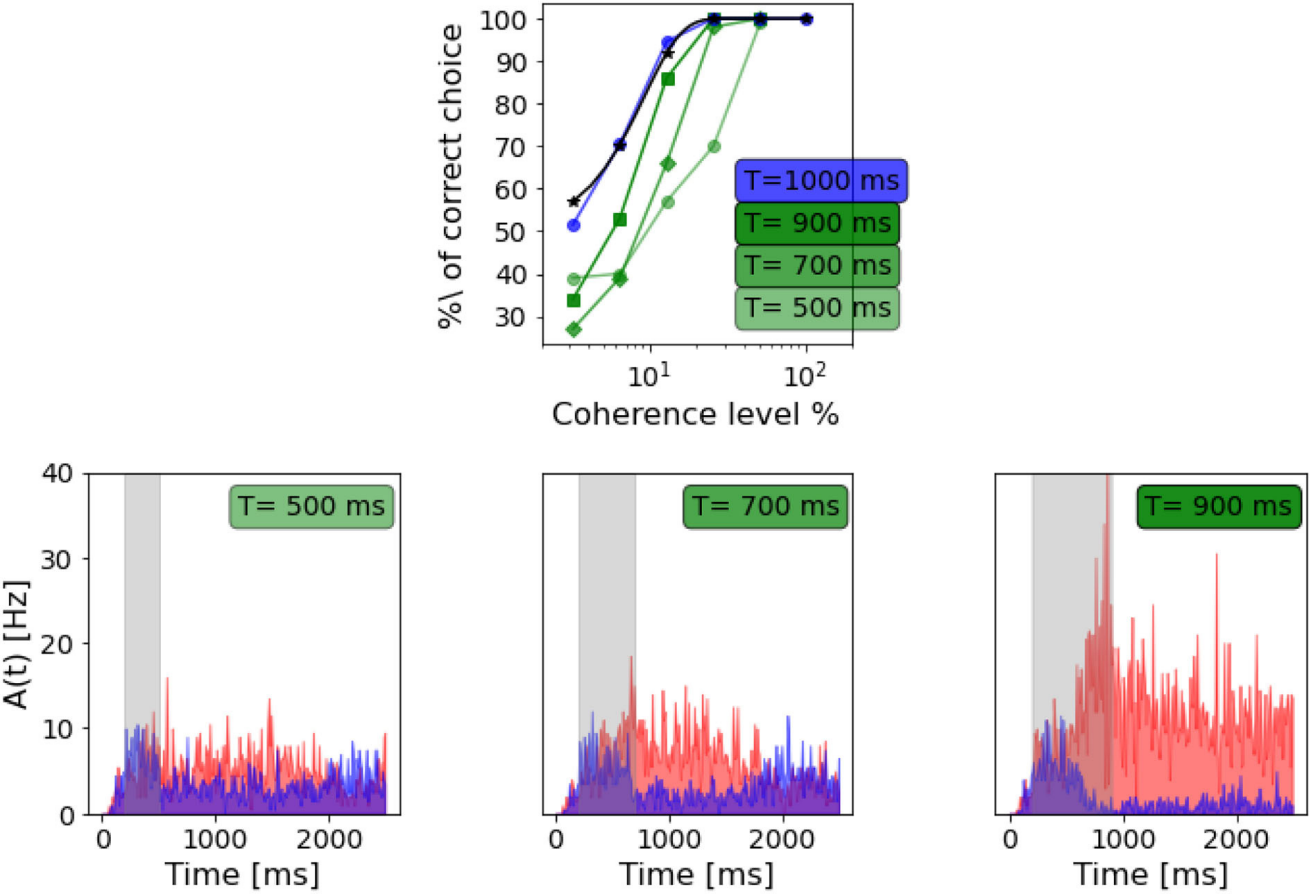
Dependency on stimulus duration: Top panel: neurometric function for different stimulus duration compared to the weibull fit as reported by Wang (2002) (black curve). Bottom panels: population A and population B firing rate activity in three different trials, but same coherence level (-12.8%). From the left: stimulus delivered for 500 ms, stimulus delivered for 700 ms, stimulus delivered for 900 ms.

Britten et al. (1992) tested the dependency of the decision-making process on the duration of the stimulus, i.e., how long the stimulus should be to formulate a net decision. We tested the network's performance when varying the stimulus duration, as shown in Figure 8.

As in Wang (2002), we also found that decreasing the stimulus duration decrease the ability of the network to take a decision. In Figure 8, we can see that with a stimulus of 700 ms, the network is still able to accumulate evidence and engage in a winner-take-all mechanism, although the persistent activity during the delayed period seems to be affected. When a 500 ms stimulus is delivered, this no longer stands, and even if population A has a slightly higher rate after stimulus delivery, we cannot claim that the activity of the two populations diverges significantly.

**Figure 9:**
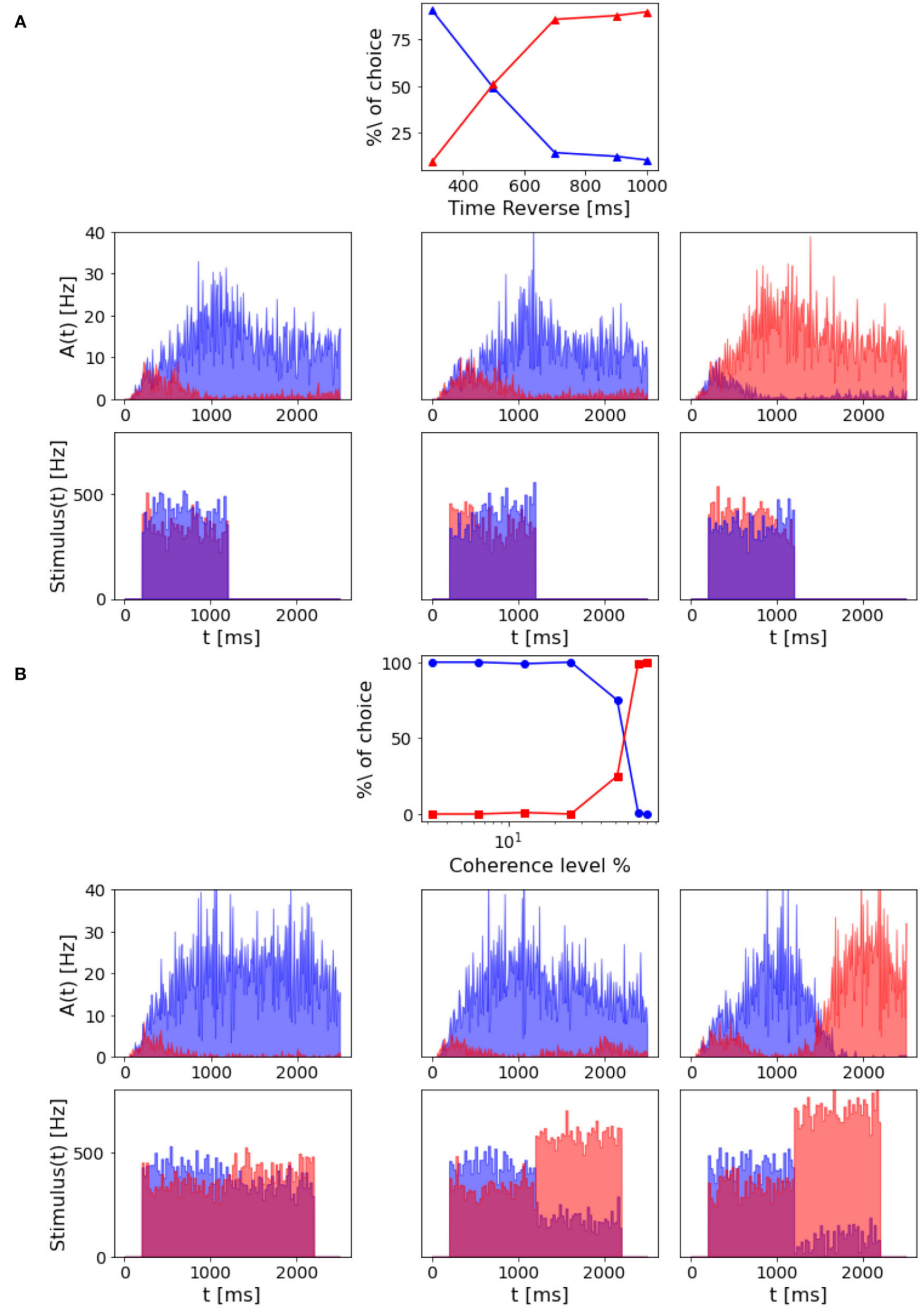
Decision reversal: **(A)** Time for stimulus reversal. Top panel: Percentage of choice for population A and population B with a –6.4% coherence stimulus in input, that is reverted at a different time (300, 500, 700, 900, and 1,000 ms). The reverted stimulus coherence is 6.4%. Central panels: population A and population B firing rate activity in three different trials where the stimulus is reverted, after 300 ms (left), 700 ms (central), and 900 ms (right). Bottom panels: Corresponding input rates over time. **(B)** Intensity of stimulus reversal. Top panel: Percentage of choice for population A and population B with a 12.8% coherence stimulus in input that is reverted after 1,000 ms with reverse stimuli at different intensity (–3.2, –6.4, –12.8, –25.6, –51.2, –70, and –80%). Central panels: population A and population B firing rate activity in three different trials where the reverted stimulus intensity is –12.8% (left), –51.2% (central), and –80% (right). Bottom panels: Corresponding input rates over time.

In Wang (2002), the author questions whether the network model is able to subtract evidence given a reverted signal and to accumulate evidence for the initial input signal. Thus, we also investigated this property in our model by running simulations where the input signal was reversed during stimulation, varying the stimulus reversal time and intensity. To test the time of reversal, the stimulus coherence is set to –6.4% before and +6.4% after the reversal. From Figure 9A, we can see that if the reversion happens after 800 ms, the network reaches the attractor dynamics. Thus, there are no effects on behavioral decision performance. While, if the reverse stimulus onset is early enough, the percentage of choice for the population A depends on the time of reversal.

However, we can still reverse the behavioral outcome after 1 s stimulus if we provide the network with a stronger reverse stimulus for a prolonged time interval. As we can see from Figure 9B, when the reverse stimulus is above 70% coherence level, the network accumulates enough new evidence to change the previously made decision.

### 3.5. Virtual behavioral task analysis

**Figure 10:**
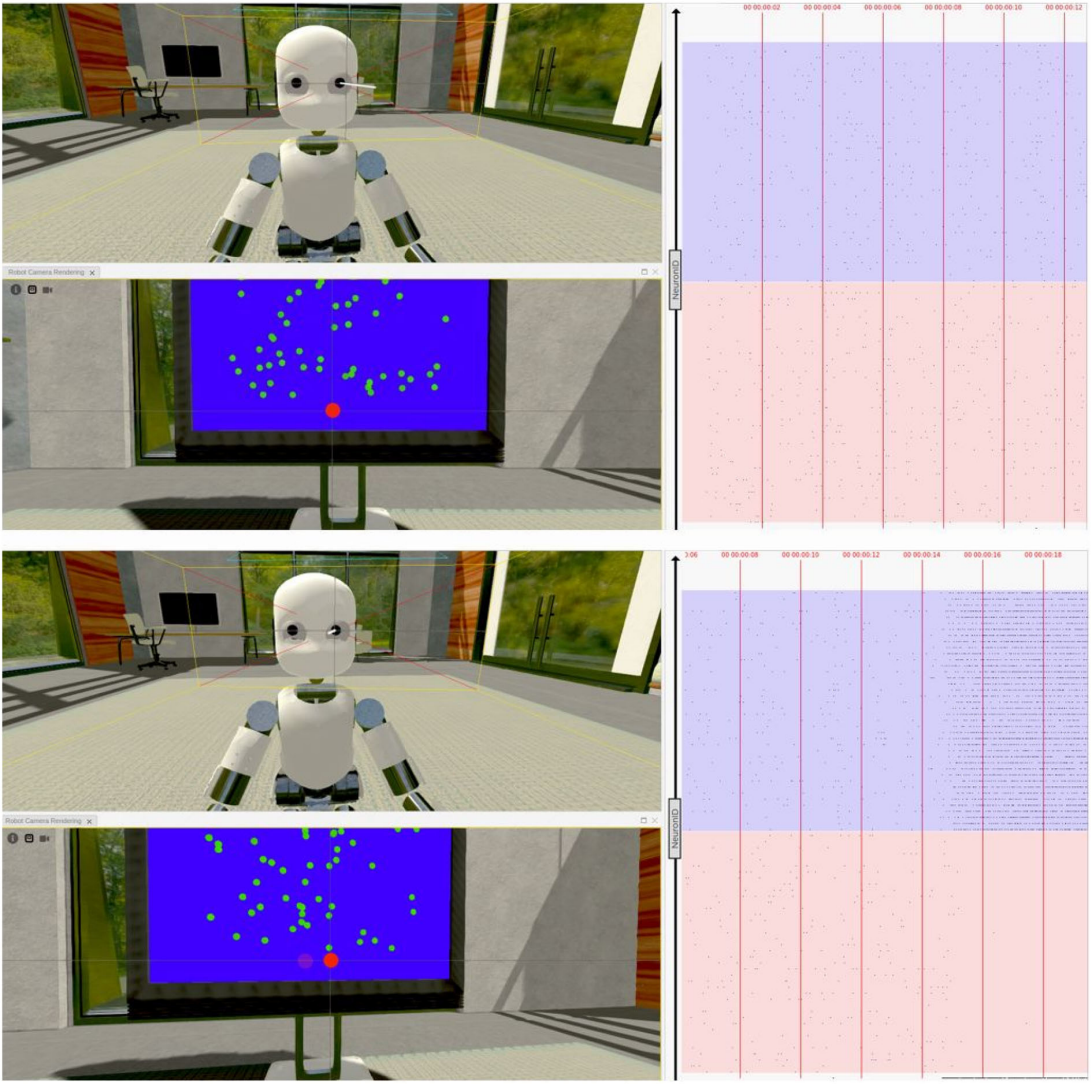
Virtual task execution: Here, reported two screenshots of the NRP interface during the first 20 s of a virtual experiment execution. The iCub robot successfully performs a saccadic movement from its fixiation point (top) to the right (bottom), when shown random moving dots with a coherence value of 51,2%. The 3D renderings show both a frontal view of the iCub with its left camera frustum and the subject's field of view in which a red dot indicates the gaze target; the raster plot on the right is updated in real-time and shows the activity of the brain model embedded in the robot, marked with the corresponding timestamps. Note that, when using the NRP, the raster plot slides from right to left, thus the instantaneous value is only depicted on the rightmost edge and only past activity values are shown on screen. For the sake of clarity, the raster plots depicted in the figure only show a subset of excitatory neurons, and colored boxes have been overlaid to mark rows belonging to different populations (in red those of population A and in blue those of population B); it can be seen that starting from a balanced state (top), the network engages in a winner-take-all competition upon stimulus onset (bottom), after the 14 s mark.

We carried out different experiments on the NRP to test the ability of the network model to let a virtual robotic subject perform a visual discrimination task once embedded in a sensory-rich environment. Indeed, the iCub robot, while controlled by the excitatory population firing rates, was able to correctly perform saccadic movements following the direction of the coherently moving dots (Figure 10).

We were also able to verify that the network's activity closely matched that of its NEST implementation when running on a different software tool (Figure 10), thanks to a real-time raster plot displayed on the NRP interface. Then, to validate the simulated experimental outcomes, we tested whether the robotic subject's eye movements speed changed in relation to stimulus coherence levels by recording and analyzing the angular value of the joint controlling the iCub camera rotation when changing the stimulus strength. We considered the binary decision taken once the angular rotation of the camera joint surpassed a threshold value of 0.08 rad (4.6°) from a starting position of 0°, after the subject was presented with the stimulus.

**Figure 11:**
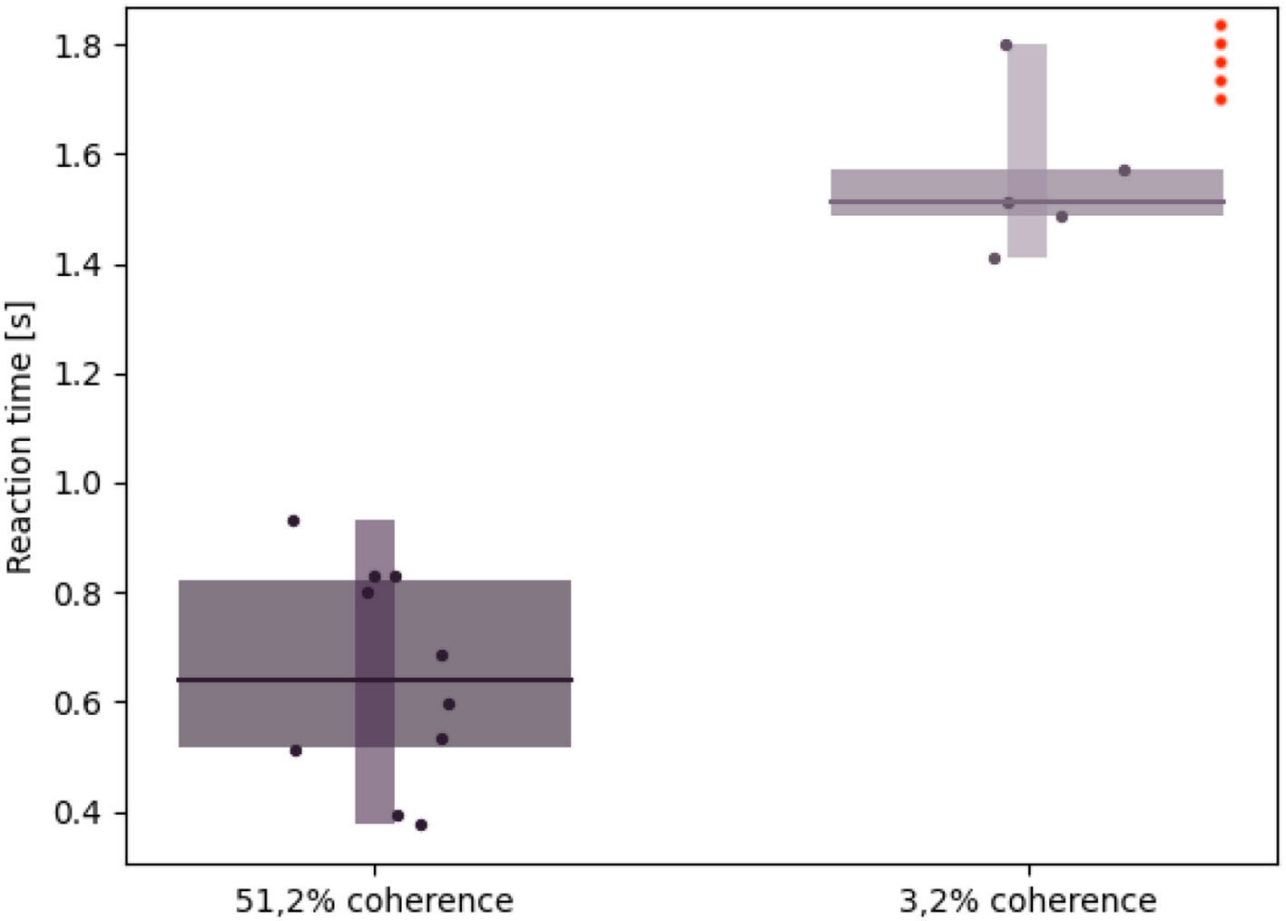
Robotic subject's reaction times: Whisker plot showing the reaction time (i.e., the time needed by the iCub to rotate its cameras by an angle greater than 0,08 rad after the stimulus onset) during 10 different trials and with different stimulus coherence values. The red dots on the upper right corner represent failed trials, in which either the reaction time exceeded 2 s or the saccadic movement didn't surpass the threshold (hence, no decision was taken).

As seen in Figure 11, higher coherence values correspond to faster reaction times, in accordance with *in vivo* visual discrimination tasks (Britten et al., 1996; Shadlen and Newsome, 2001; Roitman and Shadlen, 2002). The average reaction time during low coherence trials is more than two times the one observed with high coherence stimuli.

Furthermore, a coherence value close to 0% results in failed trials, e.g., saccade movement lower than the threshold value or reaction times exceeding more than 2 s. While this might be due to a fuzzier optical flow computation as a result of almost no coherently moving dots, it also mirrors the primate subjects' uncertainty when presented with ambiguous stimuli.

## 4. Discussion

This paper proposes a NEST implementation of Wang (2002) decision-making network model and challenges it in a virtual visual discrimination task on the NRP. We performed multiple simulations to assess its equivalence with the original study and validate its ability to perform an *in silico* behavioral task when embedded in a digital environment.

We show that our implementation of Wang (2002) model reproduces the salient features of LIP neurons during a visual discrimination behavioral task (Shadlen and Newsome, 2001; Roitman and Shadlen, 2002). The network response to the stimulus is characterized by an initial slow increase in the firing rate as a consequence of the progressive accumulation of evidence that will eventually form the final decision. We also succeed in replicating the dependency between the coherence level and the steepness of the ramping activity: the higher the coherence level, the faster the ramping response.

Another fundamental mechanism needed for having two different behavioral outputs due to the two opposite choices is the winner-take-all competition and the persistent activity after the stimulus ends. The winner-take-all competition allows one excitatory population to progressively silence the other when the decision is formed, see Figure 3. This has been achieved, thanks to the inhibitory feedback loop that suppresses the activity of one population over the other. The persistent activity is obtained due to the strong recurrent weights within a population and the presence of the slow dynamics of NMDA receptors. The network would escape the attractor state without one of the two contributions as soon as the stimulus terminates (see Figure 5). In particular, when we decrease the contribution of recurrent excitatory weights, the network still performs the winner-take-all competition in case of a strong level of coherence in input. However, it loses the ability to sustain its activity for a delayed period of time, indeed losing the working memory property of the model. In Wang (2002), when only fast AMPA receptors mediate the excitatory connections, the network is still able to reach attractor dynamics but cannot integrate the stimulus for more than 10 ms. In our implementation, when we reduce the NMDA receptor dynamics to be the same as AMPA receptors, the network loses the ability to ramp up, i.e., integrate the stimulus for a longer time span, as in Wang (2002). However, then it never reaches a sufficient firing rate to engage a strong inhibition on the opposite population. Thus, our network also loses the ability to engage the winner-take-all competition between the excitatory populations, along with the ramping activity and the persistent activity after the stimulus ends.

With the “*coin toss*” simulation, we verify that the network is not a mere stimulus amplifier because it correlates with the outcome of the decision and not with the input stimulus coherence. Moreover, with a low coherence level, we would still observe strong responses from the non-preferred population in some trials, as observed in the experimental protocol in Britten et al. (1996); Shadlen and Newsome (2001), and Roitman and Shadlen (2002).

All the major differences in the results between our implementation and the one proposed by Wang (2002) could be due to a non-optimized tuning of the network parameters. Indeed, we experience some difficulties replicating the network with the identical model architecture since the network's activity is often saturated (all neurons spiking at high-frequency rates) or shows oscillatory behavior, even without stimulation. This is the main reason for not implementing a fully connected network and instead using a pairwise Bernoulli probability to set the connection degree between and within a population. Thus, a further investigation of the optimal parameters for the network parameter could be done using *ad hoc* genetic algorithms and a straightforward comparison with experimental data.

The neuro robotics platform is a software tool publicly available within the EBRAINS infrastructure that can be used by any researcher with minimal software programming knowledge. It leverages its learning curve for new users by providing simple templates to edit and reverse engineer. Here, we show a more sophisticated use-case of the platform. We demonstrate that on the NRP, it is possible to implement *in silico* replicas of behavioral experiments deeply investigated in literature (Britten et al., 1996; Shadlen and Newsome, 2001; Roitman and Shadlen, 2002).

The proposed visual discrimination task reproduction is based on our NEST implementation of the decision-making network but required us to design both the experimental setting and stimulus delivery in accordance with biological mechanisms. To do so, we chose an iCub humanoid robot as our subject since its behavioral repertoire covers the saccadic movements needed for the experiment. Then, we replicated the stimulus delivery method commonly used in analogous primate studies, namely a screen displaying random moving dots with varying coherence levels. Finally, we implemented a transfer function able to encode stimulus coherence levels from the images seen by the subject, akin to biological perception.

Although the transfer function we designed successfully allowed the robotic subject to perform the visual discrimination task, the optical flow is estimated from the iCub cameras using a relatively simple, yet well-established image processing algorithm (Lucas and Kanade, 1981). This design choice was mostly due to avoid introducing a delay between the onset of the visual stimuli and the network inputs by using an optimized implementation of the algorithm in OpenCV (Bradski, 2000), a real-time computer vision library already integrated into the NRP.

Using more recent and sophisticated saliency detection methods (as those proposed in Jian et al., 2018, 2021) could increase the precision of the encoded visual stimulus, but their feasibility of implementation and optimization within the intricate architecture of the NRP shall be further investigated further.

Finally, just as in our reference study (Wang, 2002), we implicitly model many visual pathway structures and only mimic the neural representation of the stimulus through a Poisson generator with varying firing rates. Moreover, we employ a simplified mechanism to convert the two spiking population firing rates to saccadic commands by imposing a rotation of the robot's camera joint by a value proportional to the network activity. Although these mechanisms did not represent the scope of the present study, they still represent a structural difference between the primates experiment and our virtual replica.

However, we believe that, by sharing our implementation with the scientific community, such mechanisms could be faithfully reproduced by other research groups, ultimately leading to a more realistic investigation of the biological mechanisms underlying a visual discrimination task.

## Data availability statement

The datasets presented in this study can be found in online repositories. The link of the repository can be found below: https://github.com/alessandratrapani/decision_making_NEST_NRP.

## Author contributions

AT and FS designed the computational framework and analyzed the data. VG performed the first implementation of the network model. AT, FS, EB, SC, LC, MD'A, FD, FF, and VF carried out the final implementation and performed the simulations. AT and FS wrote the manuscript with input from all authors. AP conceived the study and was in charge of overall direction and planning and revised the manuscript. All authors contributed to the article and approved the submitted version.

## Funding

This project/research has received a Voucher (CEoI 4- Rodent microcircuits: RisingNet Whole-bRaIn rodent SpikING neural NETworks) from the European Union's Horizon 2020 Framework Programme for Research and Innovation under the Specific Grant Agreement No. 945539 (Human Brain Project SGA3).

## Conflict of interest

The authors declare that the research was conducted in the absence of any commercial or financial relationships that could be construed as a potential conflict of interest.

## Publisher's note

All claims expressed in this article are solely those of the authors and do not necessarily represent those of their affiliated organizations, or those of the publisher, the editors and the reviewers. Any product that may be evaluated in this article, or claim that may be made by its manufacturer, is not guaranteed or endorsed by the publisher.
